# Supplementary material for: Saliency-Aware Subtle Augmentation Improves Human Visual Search Performance in VR
Source: Brain Sci. 2021 Feb 25;11(3):283. doi: 10.3390/brainsci11030283 (PMC7996609; doi:10.3390/brainsci11030283)
Supplement: Supplementary file 1 [file brainsci-11-00283-s001.pdf]

Supplemetary Information

S1 Search target locations

Prior to the experiment, a set of nine possible target locations (ϕ, θ) was generated for each visual scene, where ϕ could be in the range [0°, 135°] and [225°, 360°], and θ in the range [45°, 135°]. The distribution of the final set of possible search target locations for all visual scenes is shown in Fig. S1.

S2 Visual scenes used in the training phase

In Fig. S2 five visual scenes are shown which were used during the training phase.

S3 Visual scenes used in the main experiment

In Fig. S3 and Fig. S4 24 visual scenes are shown which were used during the main experiment. The scenes are demonstrated as the original images, not blurred.

S4 Saliency maps

In Fig. S5 and Figure S6 the saliency maps are shown which were used to blur images for the main experiment. Each saliency map corresponds to the respective image in Figure S3 and Figure S4.

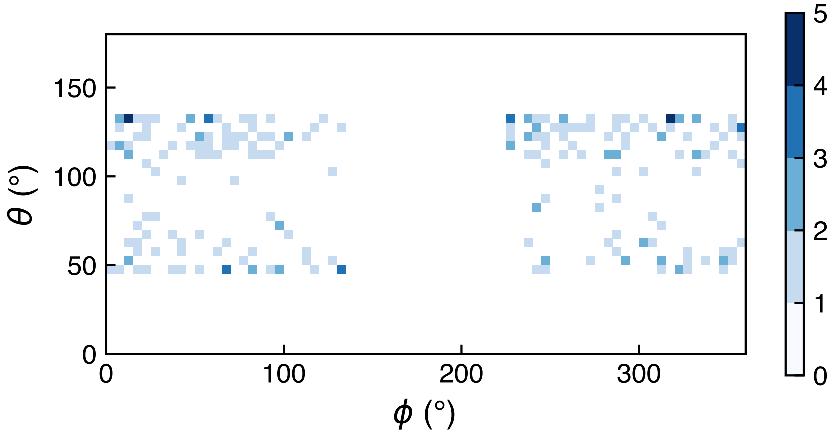

Figure S1: Distribution of possible search target locations for all visual scenes in spherical angle coordinates. For each scene nine possible location were randomly selected within regions with saliency values under 17% of maximum saliency value, and where ϕ is in the range $[0°, 135°]$ and $[225°, 360°]$, and θ is in the range $[45°, 135°]$. The distribution is shown as a 2D histogram with bin size $5°$.

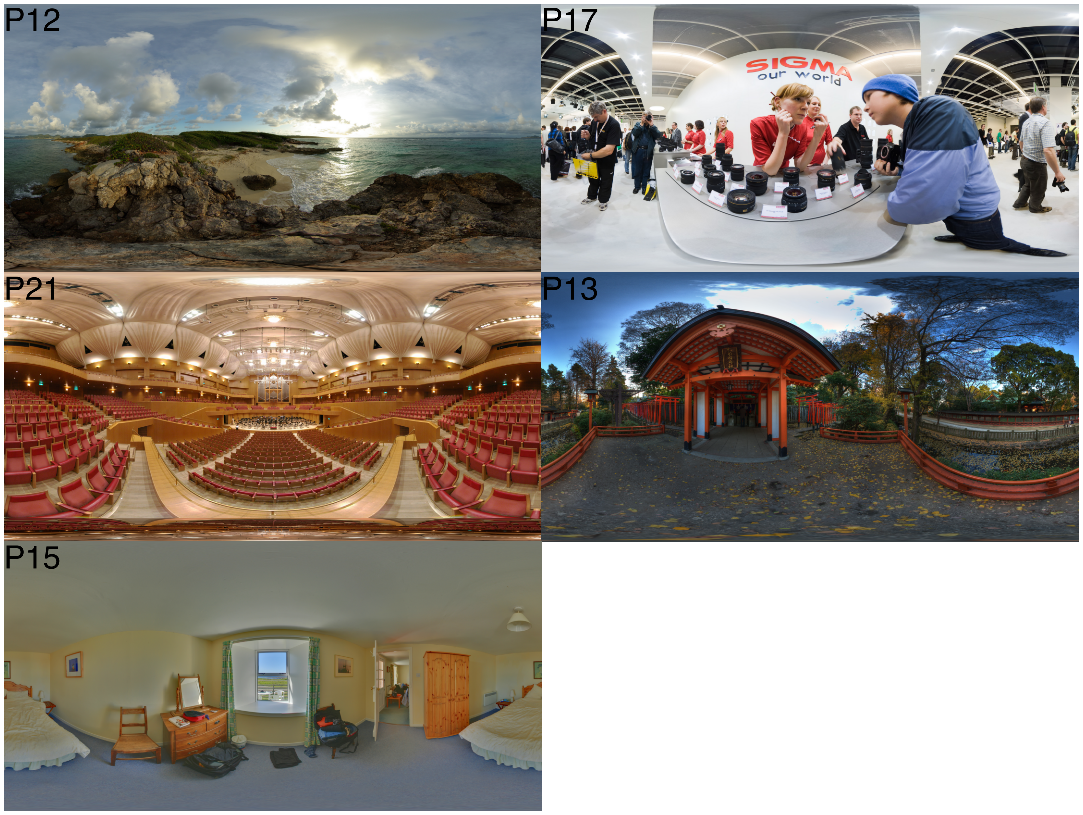

Figure S2: Five visual scenes used during the training phase. In the upper left corner of each scene the indicator of the image is placed, which represents the name of the file in the Salient360! Training dataset.

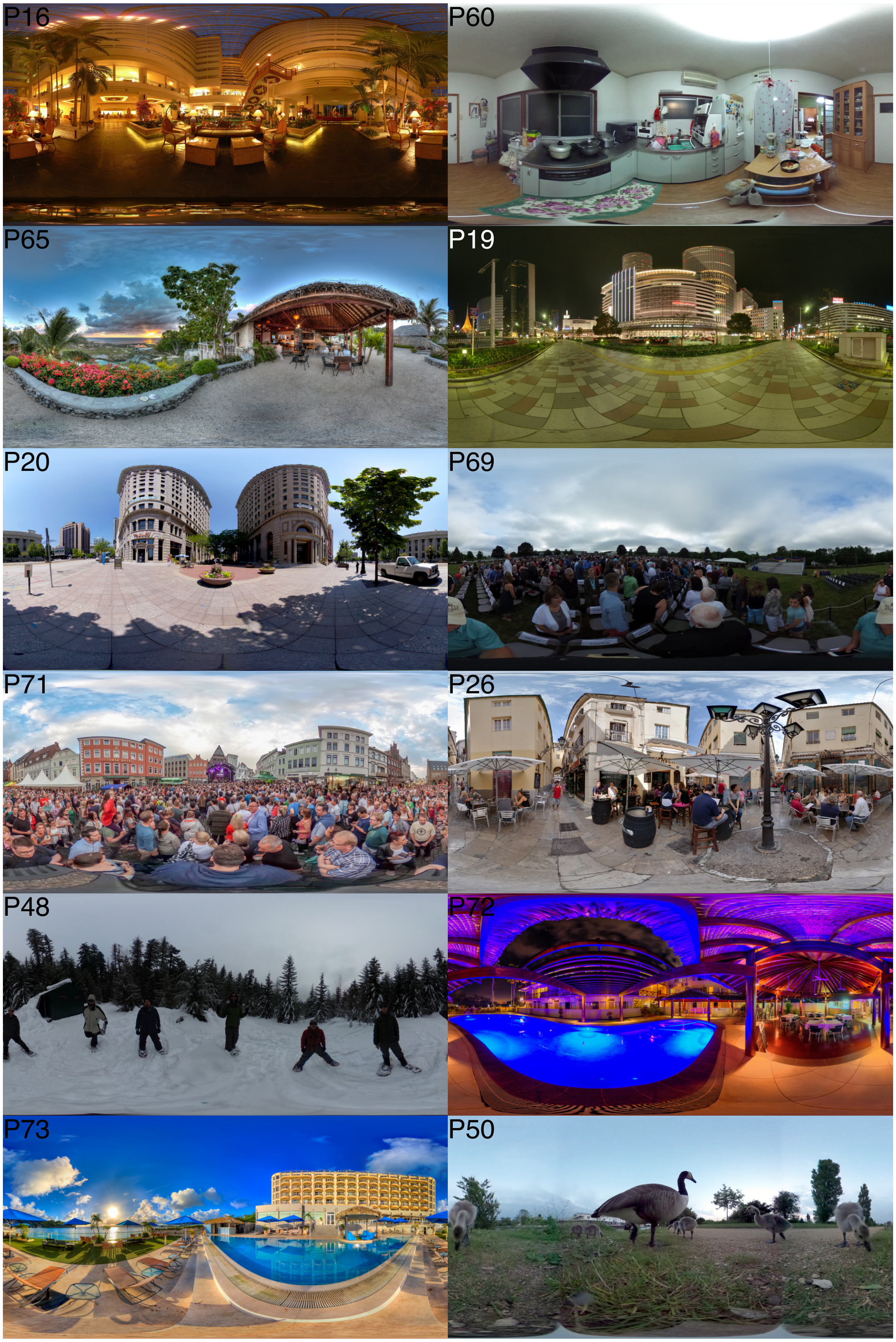

Figure S3: The first half of the 24 visual scenes used during the main experiment. In the upper left corner of each scene the indicator of the image is placed, which represents the name of the file in the Salient360! Training dataset.

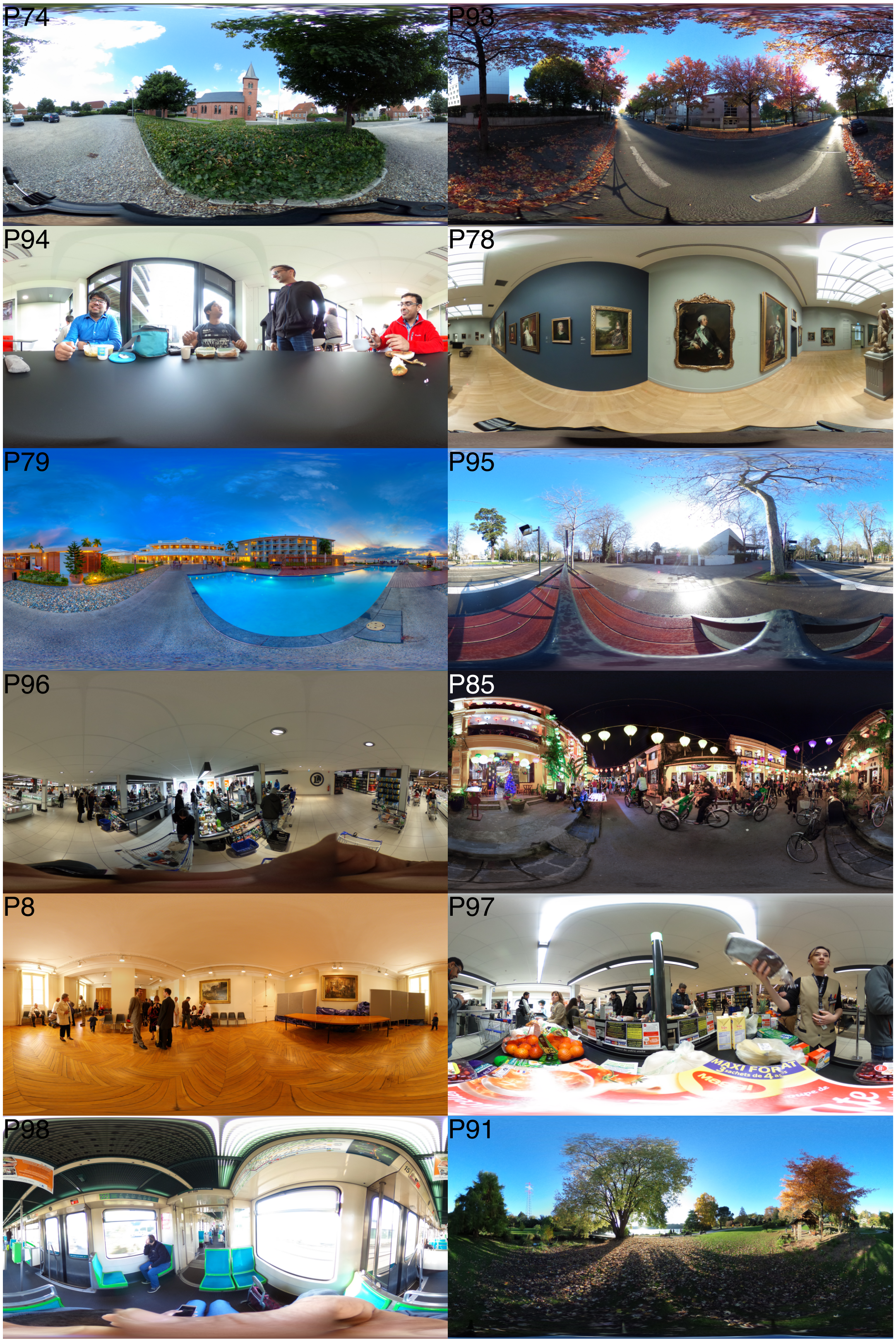

Figure S4: The second half of the 24 visual scenes used during the main experiment. In the upper left corner of each scene the indicator of the image is placed, which represents the name of the file in the Salient360! Training dataset.

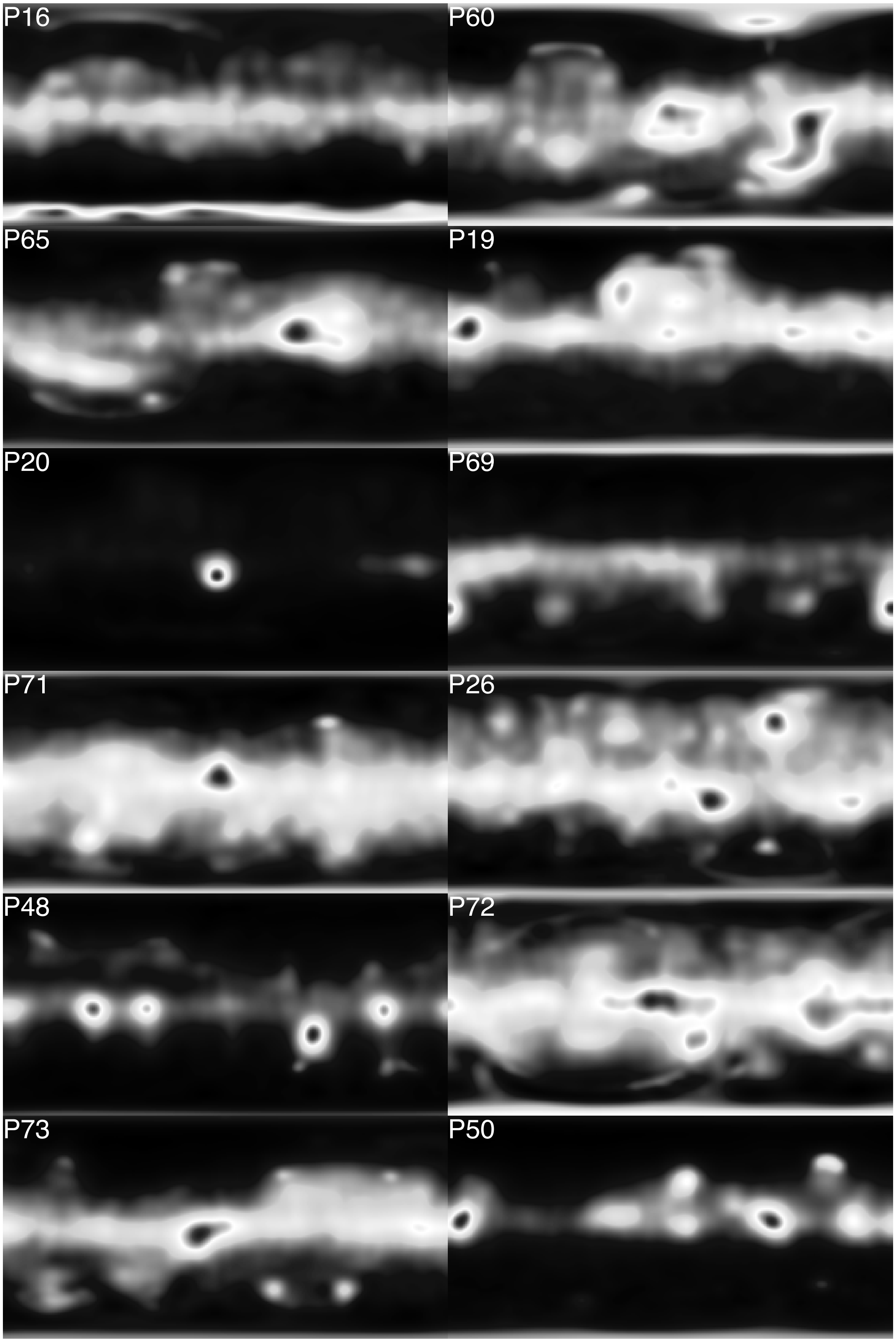

Figure S5: The first half of the 24 saliency maps in gray scale used to blur images for the main experiment. In the upper left corner of each map the indicator of the image is placed, which corresponds to the respective image.

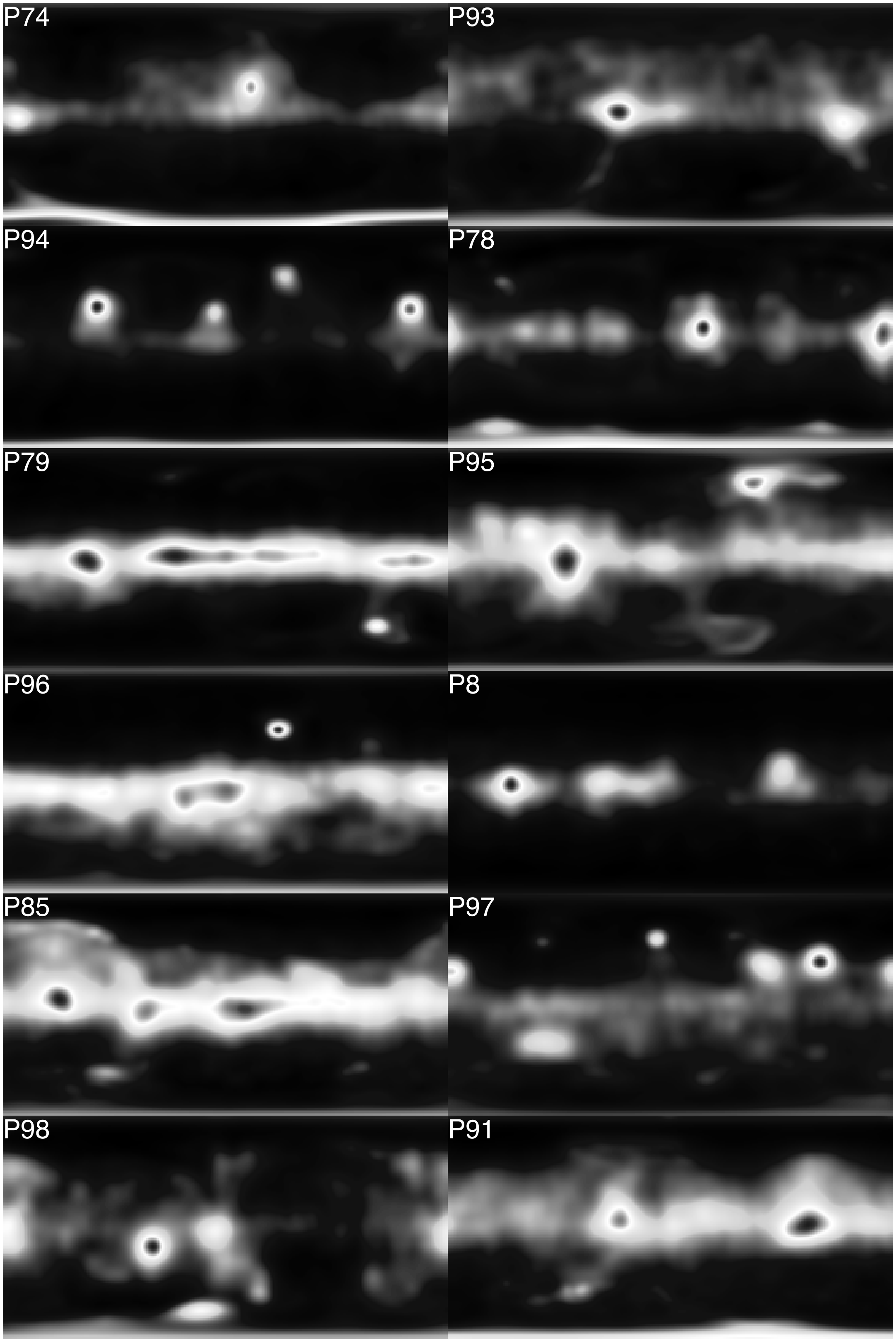

Figure S6: The second half of the 24 saliency maps in gray scale used to blur images for the main experiment. In the upper left corner of each map the indicator of the image is placed, which corresponds to the respective image.

S5 Individual results for behavioral metrics

In Figure S7 and Figure S8 the individual results for the search time and the proportions of trials where the search target was not found, are shown for each of twenty subject.

S6 Individual results for fixation metrics

In Figure S9, Figure S10, and Figure S11 the individual results for $NumOfFix$, $PropFixInAOI$, and $FixDuration$ (see the main text) for each participants are shown.

S7 Learning

In Figure S12 the evolution of mean search time, proportion of missed trials, as well as proportion of fixations in the area of interest ($PropFixInAOI$) are shown across different blur conditions over the time of the experiment averaged among all subjects. Each block contained identical number of trials (see main text). A general learning of the task is observed for all three blur conditions which is indicated by a general downtrend for all metrics as a function of the block number. This is expected, as most of participants get more comfortable with the headset across the time of the experiment, as well as get familiar with the visual scenes which are identical 24 visual scenes in each block. However, the difference in performance metrics and $PropFixInAOI$ between the no-blur condition and other two blur conditions is apparent already in the first block of the experiment, particularly, for the proportion of missed trials and $PropFixInAOI$. See the discussion in the main text.

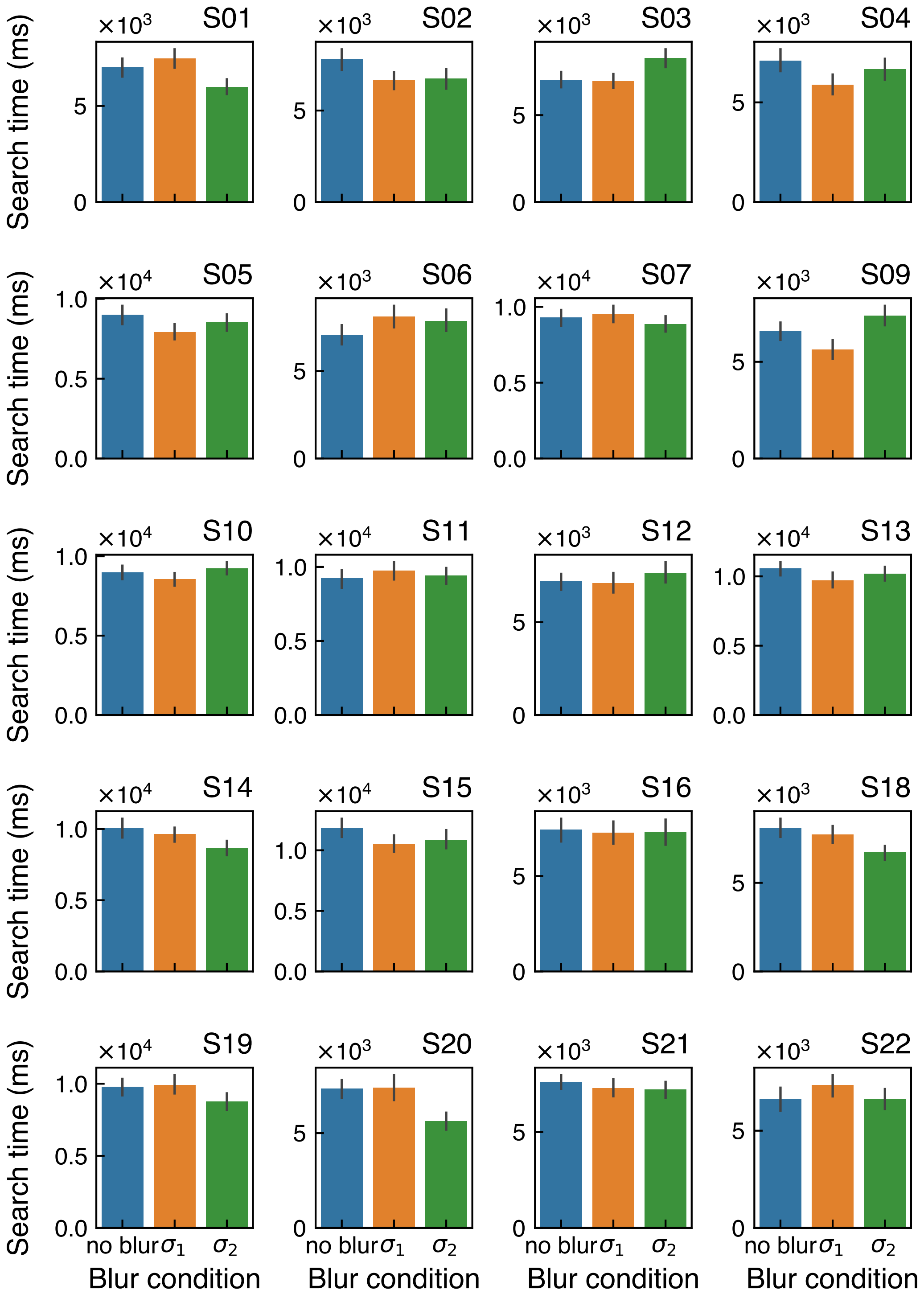

Figure S7: The individual results for the search time across different blur conditions in the trials where the target was found. The values range between 0 and 20 000 ms which is the time limit for the search. The error bars correspond to the standard error of the mean across all trials within each subject. In the top-right location of each individual subplot a pseudonym of the participant is indicated.

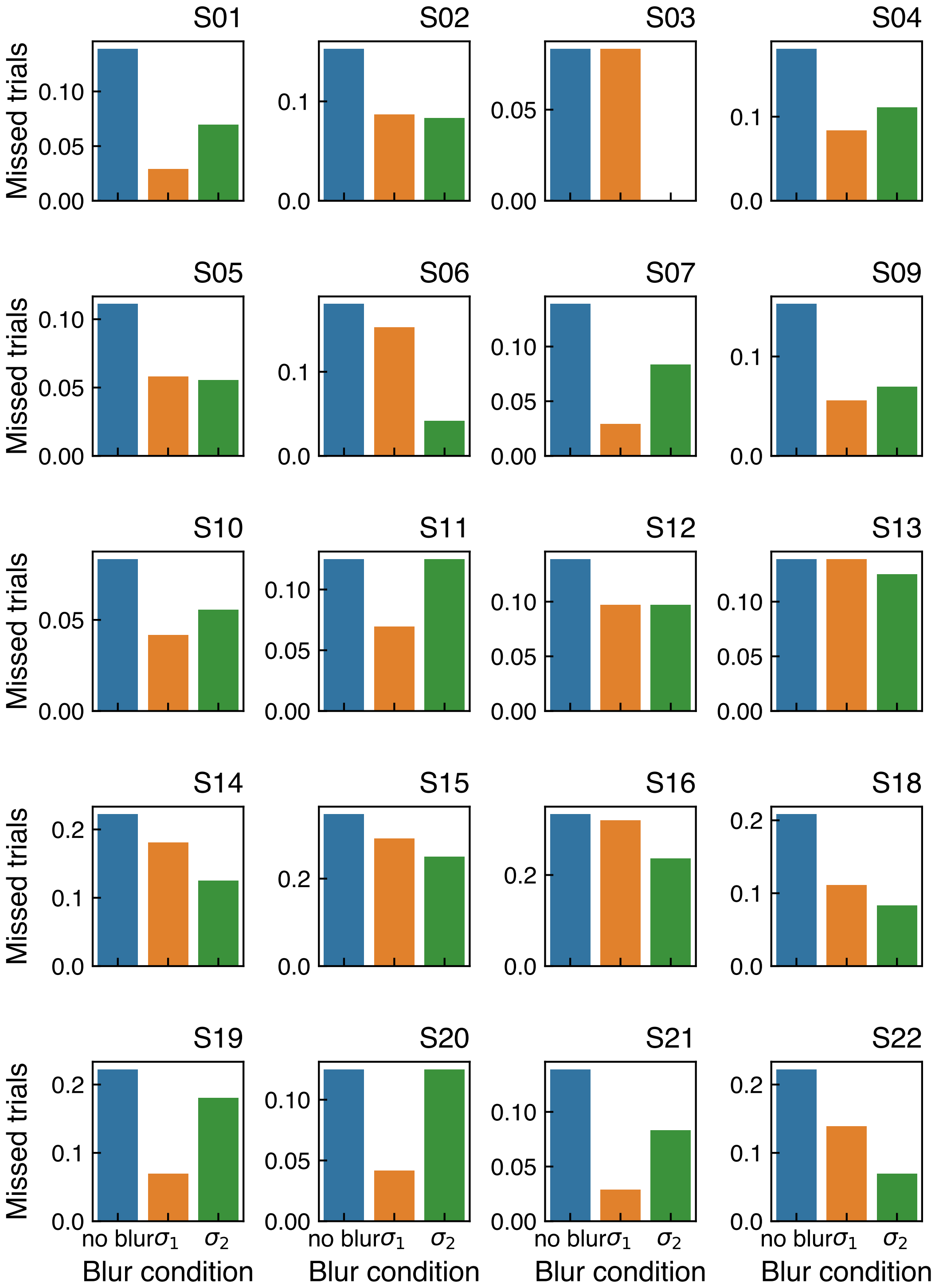

Figure S8: The individual results for the proportion of trials where the search target was not found across different blur conditions. The values range between 0 and 1 with a minimum step of 0.014. In the top-right location of each individual subplot a pseudonym of the participant is indicated.

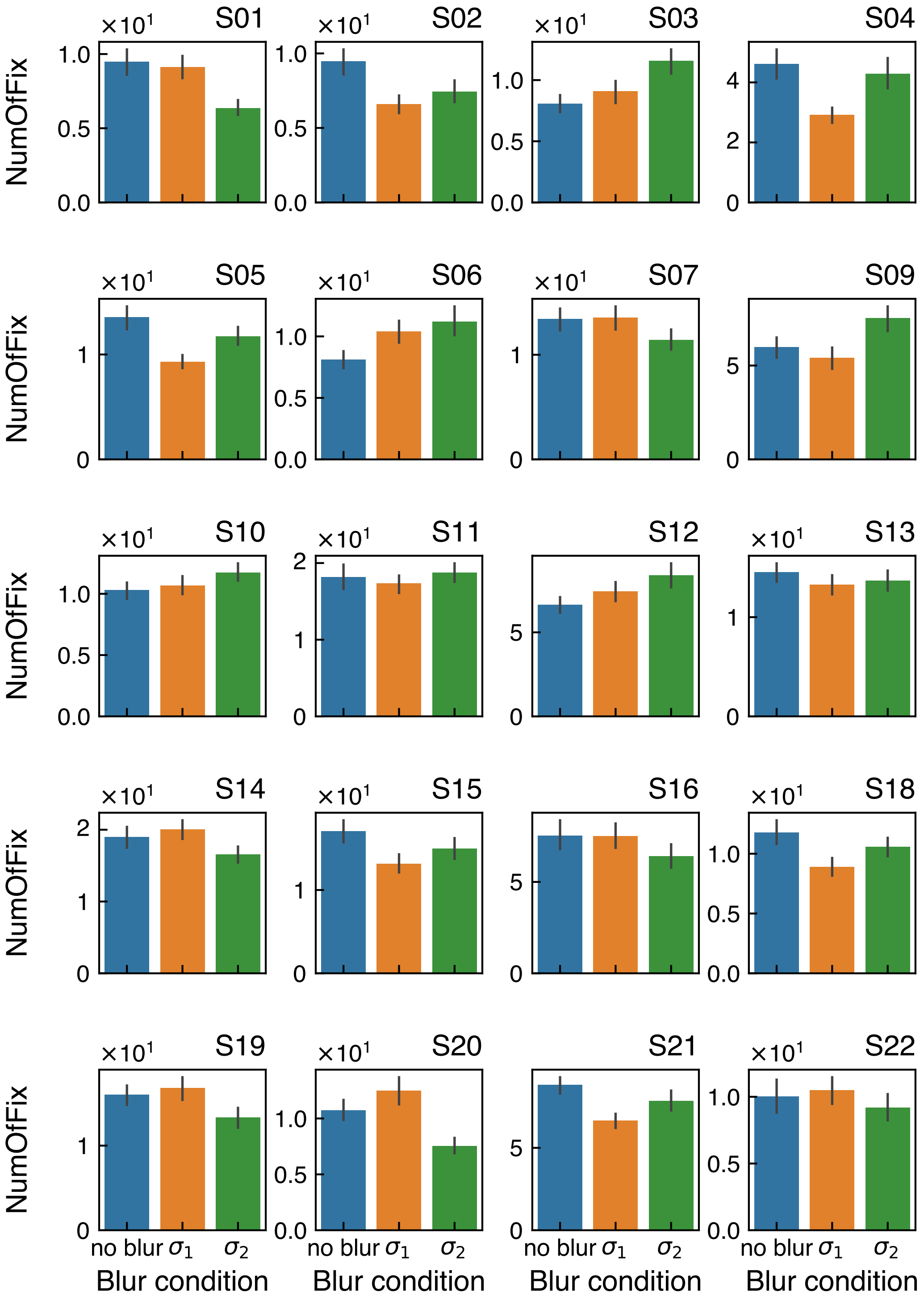

Figure S9: The individual results for the number of fixations ($NumOfFix$) in the trials where the target was found across different blur conditions. The error bars correspond to the standard error of the mean across trials within each subject. In the top-right location of each individual subplot a pseudonym of the participant is indicated.

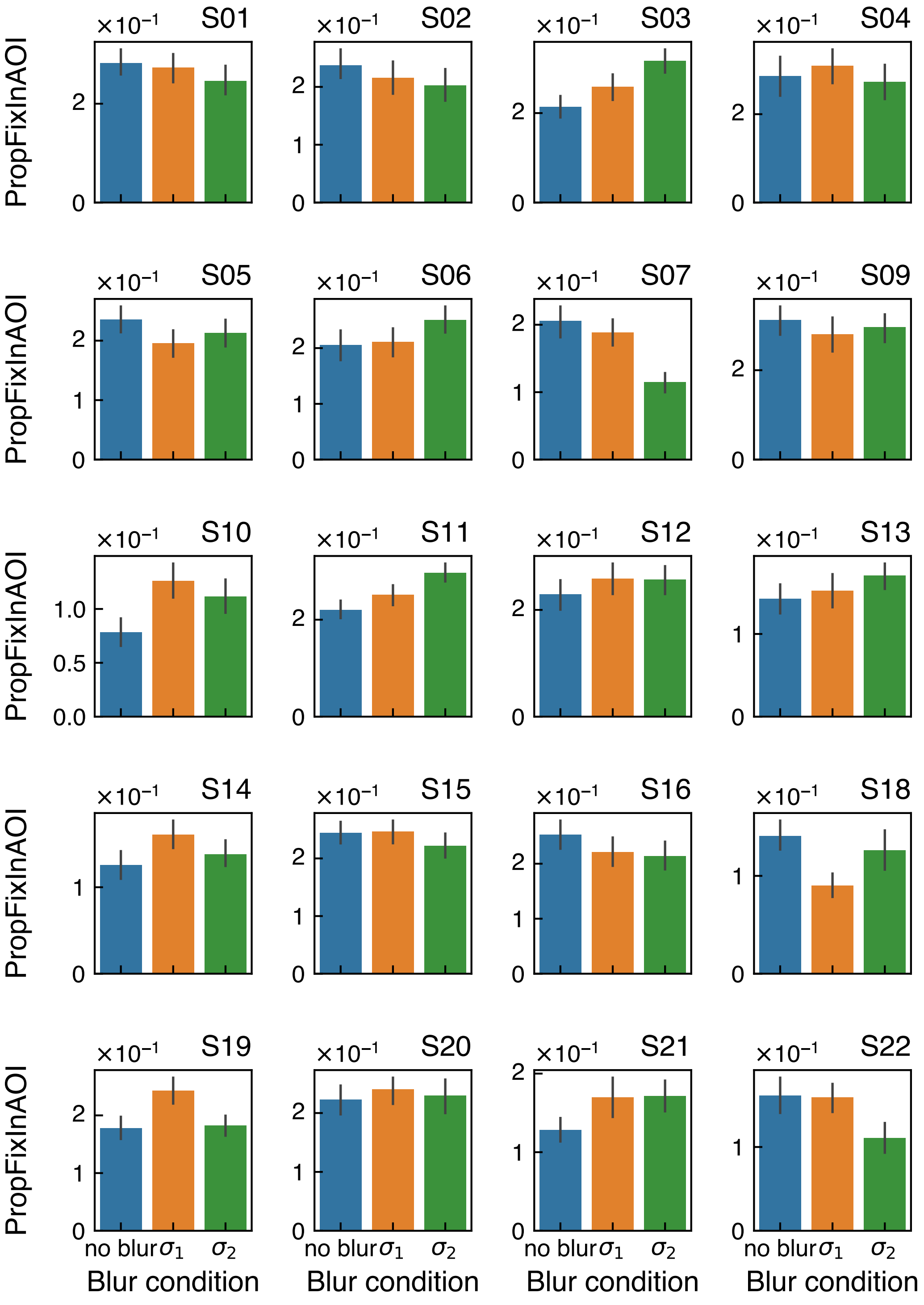

Figure S10: The individual results for the proportion of fixations within the area of interest ($PropFixInAOI$) across different blur conditions. The error bars correspond to the standard error of the mean across trials within each subject. In the top-right location of each individual subplot a pseudonym of the participant is indicated.

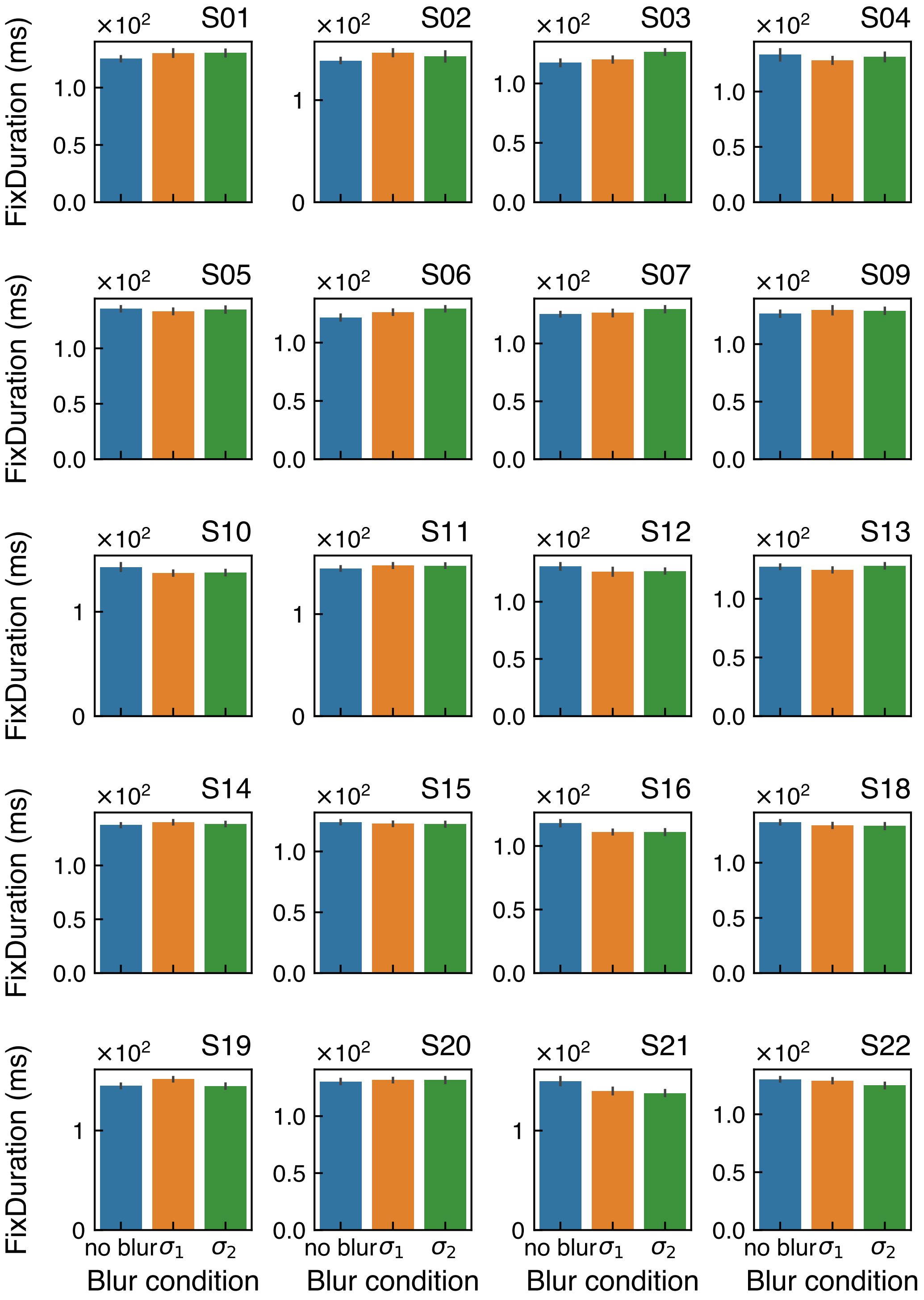

Figure S11: The individual results for the fixation duration ($PropFixInAOI$) across different blur conditions. The error bars correspond to the standard error of the mean across trials within each subject. In the top-right location of each individual subplot a pseudonym of the participant is indicated.

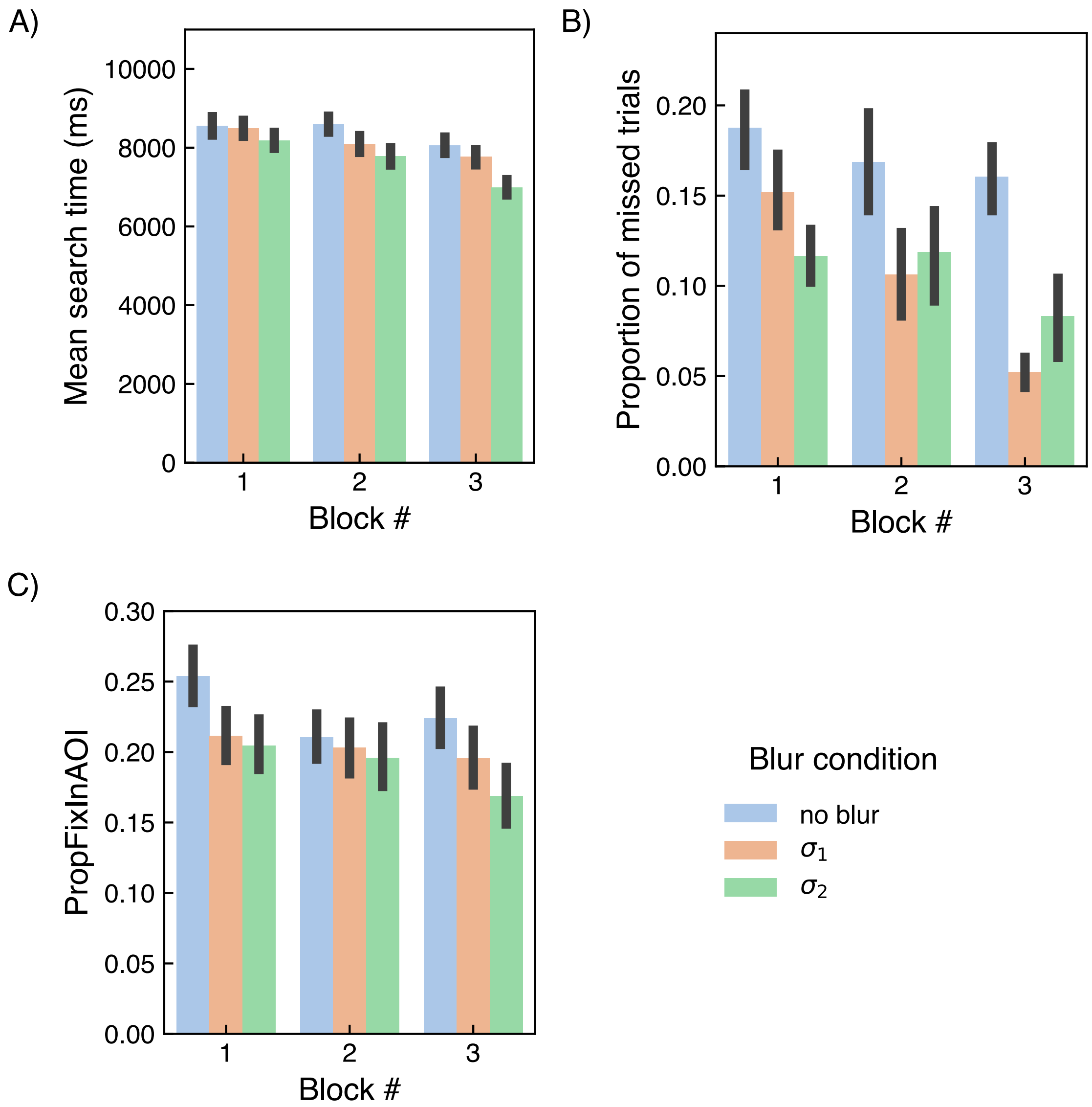

Figure S12: The evolution of mean search time, proportion of missed trials, as well as proportion of fixations in the area of interest ($PropFixInAOI$) across different blur conditions over the time of the experiment. The hue of the bars correspond to the blur condition. Each block contained 72 trials where the number of trials for each blur condition was identical (see main text). The number of the block corresponds to the order in time when the block was performed. The error bars correspond to the standard error of the mean
